# Care of the Consumptive

**Published:** 1907-08

**Authors:** Louis C. Rouglin

**Affiliations:** Atlanta, Ga.


### CARE OF THE CONSUMPTIVE.*

By Louis C. Rouglin, M. D., Atlanta, Ga.

---

If an apology is necessary for presenting for your consideration a subject so well worn as the care of the consumptive, the profusion of literature and discussions on this subject which tend to confuse and bewilder us, demonstrates the necessity for a review of the subject from time to time, and the need of separating those which are beneficial and practical of value, from the merely theoretical and speculative views, and I propose to present for your consideration and discussion only such measures whose benefits and practical value have been thoroughly demonstrated.

### PRIVILEGE AND DUTY OF THE PHYSICIAN.

It is our privilege as physicians to relieve suffering and prolong life, but it is our sacred duty to prevent disease. The community and our patients look to us in all matters pertaining to health, and desire to be instructed how to prevent disease. It is our duty that we as physicians should enlighten, educate, and instruct the public in matter of prophalaxis; in this manner we can achieve far greater results, for we are but too often powerless to give the desired cure when the disease has once gained a foothold,—and in no disease is this more conspicuously true as in Tuberculosis.

It is well known that Tuberculosis is a preventable disease and in a great number, especially in its incipiency it is a curable disease, yet,

---

*Read at the meeting of the American Anti-Tuberculosis League at Atlantic City, N. J., June , 1907.

200,000 valuable lives are each year cut short by this White Plague. Can we afford to make such a sacrifice? Can we permit this dread disease to continue onward and forward, spreading death, ruin and desolation in its path, unabridged and unhampered by any efforts of our own to check its murderous and deathdealing progress when proper education of the masses, proper measures of the municipality, state and patient himself can gradually diminish its mortality and in time entirely stamp out and eradicate the disease? But this cannot be accomplished simply by the successful treatment of the individual case, but only by the elimination of those vicious conditions which give rise to the disease, and it is the physician's duty to point out the way and the public-spirited citizens should unite with the profession in a concentrated and untiring effort for its elimination.

### DUTY OF MUNICIPALITY AND STATE.

It is the duty of every municipality and state to secure, for its Board of Health, men scientifically equipped to deal with the sanitary problem, men that are energetic, fearless and agressive, for it is war against ignorance and indifference; clothe them with suficient authority, give them a salary sufficient to insure their independent action and that they may be able to devote their entire and undivided time to protect the health of their citizens.

### GENERAL SANITATION.

Street sweeping should receive proper attention. Tuberculosis is properly called a "dust disease." The street sweeping machines which drive dust to the cart to be shoveled into wagons are unsanitary and a source of much danger,—under no circumstances should it be permitted to be done during the day and when pedestrians are crowding the streets. The flushing process is safe, sanitary and most effective. Rigid supervision of the cleaning of alleys and back yards should be enforced. Street cars and other public conveyances should receive attention from the Board of Health. Ordinances regulating their cleaning, sweeping, dusting and ventilating should be rigidly en-forced .

SMOKE ORDINANCE: Inhalations of minute particles of carbon coming from the chimneys of our dwellings, factories and trains irritate the lungs and is a prominent factor in predisposing to the disease,—ordinances regulating same should be strictly enforced.

Anti-spitting laws should be rigidly enforced, the subject is covered by Dr. Foster as follows:

"As to the legal restraint on the spitting it is not necessary to dwell. We know that through indiscriminate expectoration more than in any other way tuberculosis is scattered broadcast. We all know how much has already been accomplished in eliminating the evil practice that at one time was considered our chief national characteristic. Expectorating in public places should be legally prohibited and the pubic should be made to respect the law." Inspection of dairies in regard to the condition of the herds, inspection of milk, their depots and source of supply, ordinances prohibiting the sale of undrawn poultry and inspection of slaughtered cattle, meat and fish inspection should be rigidly enforced. Schools, churches, theatres and all places where people congregate should be inspected and proper sanitary ventilation, cleaning and dusting methods should be instituted. Consumptive teachers and pupils should be excluded from public schools. Our police stations, prisons, alms-houses and all other public institutions should make special provisions for the consumptives and not permit them to intermingle and spread the disease among the healthy inmates.

### SANITATION WITH SPECIAL REFERENCE TO THE CONSUMPTIVE..

The following ordinances and provisions, have all or in part, been adopted and are carried out successfully in many of our states, and in the countries of Europe. They are all practical and have proven of utmost value in eliminating the disease, but in order to obtain the full benefit from them uniform and general adoption of same is necessary and indispensable.

1. Compulsory reports and registration of all cases of pulmonary tuberculosis, as well as any other type of communicable disease.

2. All such reports to be open for inspection only to authorized officials, who at the end of the year or semi-annually should make a public report of the number of cases reported—deaths and cures and number of those under treatment.

3. Provisions for free miscroscopical and bacteriological examinations of all suspected cases.

4. Printed instructions to be furnished to patients what methods to pursue to prevent the spread of the disease.

5. On death or removal of the patient from residence, disinfection of premises by Health Board or by owner under the supervision of the Board of Health.

6. It shall be the duty of every person afflicted with tuberculosis or in attendance upon any one affiicted with it, to observe and enforce all sanitary rules and regulations for preventing the spread of the disease.

7. Upon report of recovery, patient and attendant shall be relieved from further liability to any of the above requirements.

8. Violation of any of the above to be deemed a misdemeanor and punished accordingly.

9. Establishment of tuberculosis dispensaries where patients of the poorer class should be invited, same to be provided with the trained nurse or nurses to visit the homes of the patients and to determine the mode of living, sanitary conditions and health of the other members of the family.

Those in charge should be prepared to answer all inquiries regarding tuberculosis, and to advise in a given case what is best to be done and should constitute a centre for dissemination, in the widest fashion, of information regarding prevention and treatment.

10. Establishment of sanatoria for patients who are curable and require a special regime of treatment not procurable at their own home.

11. Establishment of a home for incurables—chiefly for the interest of other persons.

### TREATMENT.

Every method of treatment which has stood the practical test has for its prime object the improvement of nutrition and the essentials for obtaining same, I believe is conceded by all authorities to consist of 1. Rest—2. Pure fresh air.—3. Food, and I desire to go on record in adding—4. Drugs and 5. Hydrotherapy. In my opinion every case presents some time during the disease a state where drugs properly chosen and judiciously used are of the utmost value, and the stimulating as well as the sedative action of hydrotherapy and its aid to nutrition when properly applied, is a factor which must not be slighted and I believe is worth thorough consideration.

REST: It is well known that in all processes of repair, best results are obtained with the part at rest, yet in applying it to tuberculosis it is only a relative term, for what in one individual would constitute complete rest may for another mean abnormal exertion—so when we insist on a patient to be at rest, we must not only consider the tubercular condition, but also the stage of disease, complicating diseases, age, occupation, and former habits of the individual. The indications for absolute rest are, fever labored respirations, and disturbed cardiac action, but in this connection we must not forget that exercise in disease as in health, promotes nutrition, aids digestion, improves the appetite, aids oxidation and increases elimination, and when the patient's condition will permit same should gradually and judiciously be encouraged.

2. PURE FRESH AIR: The one end to be sought in the choice of climate is reaction, and when this cannot be accomplished the treatment is a failure. Re-action of the skin may be attained by either extreme cold, hot or moderate temperature,—varying of course, with the individual and with the mode, time and length of the application, this accounts to a large degree why so many diverse climates have a pronounced beneficial effect on the disease; it then follows that any climate is suitable for the consumptive where the patient being continuously in the open air will get the proper re-action. Here again the individual case must be considered, for it is well known that those with cardiac complications cannot stand high altitude, while those suffering or subject to rheumatism, do badly in low, cold damp climates. Anaemic persons cannot stand a rigorous climate as well as the plethoric,—a too sudden change of climate from warm to cold is not advisable, and that the patient may return home when cured a climate as near a possible as his native own will give best resuts. We must also take into consideration that nostalgia has quite a deleterious effect on the pognosis and it is better to have the patient near his home, comfort and friends. Under no circumstances should a patient be sent away from his home and family for a long distance unless he is well provided with funds to take the proper treatment for a sufficient length of time to affect a cure.

3. FOOD: The principles here, as in all other diseases, are chiefly to improve nutrition,—milk, raw eggs, butter are the chief articles of diet and under proper conditions can be pushed to an astonishing degree, but unfortunately all patients cannot and will not do well on this diet, and we must bear in mind that in consumption the alimentary tract is prone to disturbance, and we must constantly be on guard to see that the patient gets the quantity and quality of foods that he can digest and assimilate. Each individual case must be seperately studied and diet prescribed to suit the case. It is far better to let a patient have a varied diet nutritious in character, which he can assimilate rather than stuff indiscriminately with a restricted regime of diet.

4. DRUGS: None for the disease. We have no drugs that have a specific action upon the disease proper. Serum therapy promises much, but as yet we have no specific serum. For the special symptoms as they arise anything in the Pharmacopeia, that will meet the conditions, with due regards to its effect upon digestion and nutrition.

Cod Liver oil has a nutritive value and in some cases proves a desirable, easily-digested, fatty food, but cannot be borne by a large number of patients. Cotton Seed oil has all the nutritive value of the Cod Liver oil, can be well tolerated by a greater number of patients and is more easily digested and from my short experience with same it has in my hands proven a superior oil, more palatable and patients gain weight under its administration much more rapidly.

5. HYDROTHERAPY: The chief object to be obtained is to promote re-action and improve nutrition. Hot, cold, tepid or warm water may be used according to the conditions and requirements of the individual. To obtain good results one must have a thorough knowledge of its physiological effect, mode and technic of its application and be able to determine the temperature of water required in a given case. In the hands of the unskilled and ignorant it may do more harm than good, here as elsewhere the rule holds good that to obtain best results the remedy must be applied properly.

### DUTY OF CONSUMPTIVE TO THE STATE.

It is the duty of the consumptive to himself and to the state that he rigidly enforce all rules and laws of prophalaxis and observe and carry out all regulations prescribed by his physician, for it is on the patient himself by his care and general hygienic conduct and rigid observance of prescribed measures or precaution that a cure largely depends. He owes it to himself, in order to once more become a useful member to society to protect his family, neighbors and attendants from contracting this dread disease by heeding all sanitary precautions prescribed for this conduct. He should abstain from kissing, marrying and, if a woman, from nursing while suffering from consumption.

### CARE OF THE CONSUMPTIVE AT HOME.

The consumptive may obtain the benefit of pure fresh air at home by the arrangement of a window tent, as suggested by Dr. Knopff. This can be arranged for at very little expense to the patient and serve the double purpose of giving the patient the benefit of fresh air at home and preventing the rest of the room from becoming contaminated with tubercular bearing dust particles and sputum.

The principle on which this tent is made consists of an awning attached to a wondow inside, instead of outside of a room and attached to the bed, and so arranged that air from the room cannot enter nor mix with the air from the tent.

The tent here loaned to me by the manufacturers, Messrs. Kny-Scheerer Company, is constructed of a series of four frames made of Bessemer rods and is covered with seven ounces, extra thick sail twill, properly fitted and having elongated ends, to admit of their being tucked in under and around the bedding to prevent cold air from entering the room. A little pulley is attached to the upper portion of the window and with the aid of a cord the patient on entering the bed lowers the tent over him. A celluloid window is placed in the middle of the tent, serves to make the patient feel less out-doors, also for an observation window for members of the family and nurse. In winter patient should be covered with sufficient blankets and to insure warmth and comfort, and should wear a sweater and protect his head by means of a wollen helmet or shawl when sleeping out-doors in cold weather. When in bed the patient should be provided with a flask for his sputum, a urinal and a betl by means of which he can summon the nurse or friends in the house for communication.

### CARE OF AND BY PERSONS WHO LIVE WITH THE CONSUMPTIVE AT HOME.

Here, more than elsewhere, holds good the old and ever true maxim: "Cleanliness is next to godliness." Let order and cleanliness have the first place in the conduct of your life. Strictly observe all prescribed laws of sanitation; see that your house is properly cleaned and ventilated; dry sweeping of the room should not be permitted; keep your body, hair, teeth and nails clean; wash your hands before each meal; breath through your nostrils with the mouth shut. Take exercise outside of your dwelling and accustom yourself to outdoor life in unfavorable weather. Consult your physician on the first indication of feeling unwell.

### DUTY OF ALL—CONCLUSION.

In closing I wish to emphasize again what I have endeaovred to embody throughout the length of my paper; sanitary regulations properly ordained and rigidly enforced will serve to control and stamp out the disease, but let not the error be made that same is also a cure for the individual affected; let it be impressed upon each sufferer and victim of the disease that the good results attained in well-regulated sanatoria are not due to the better air or diet the patient gets, but on the constant supervision of the patient by the skilled physicians and the proper administration of therapeutic measures suited to the requirements of the individual patient.

It is a disease affecting all classes and all communities, the rich and poor, laborer and capitalist, young and old. We all have a duty to perform in ridding the world of this pestilence. Let us all unite, it is every one's duty to enroll in this defensive warfare. Let us all work in harmony and the twentieth century can present no greater achievement to the future generations than the stamping out this present scourge on humanity—The Great White Plague—Tuberculosis.

829-830 Candler Building.

